# A Real-Time Impedance Based Method to Assess *Rhodococcus equi* Virulence

**DOI:** 10.1371/journal.pone.0060612

**Published:** 2013-03-28

**Authors:** Aleksandra A. Miranda-CasoLuengo, Raúl Miranda-CasoLuengo, Nora T. Lieggi, Haixia Luo, Jeremy C. Simpson, Wim G. Meijer

**Affiliations:** 1 UCD School of Biomolecular and Biomedical Science and Conway Institute, University College Dublin, Dublin, Ireland; 2 UCD School of Biology and Environmental Science and Conway Institute, University College Dublin, Dublin, Ireland; University of Padova, Italy

## Abstract

*Rhodococcus equi* is a facultative intracellular pathogen of macrophages and the causative agent of foal pneumonia. *R. equi* virulence is usually assessed by analyzing intracellular growth in macrophages by enumeration of bacteria following cell lysis, which is time consuming and does not allow for a high throughput analysis. This paper describes the use of an impedance based real-time method to characterize proliferation of *R. equi* in macrophages, using virulent and attenuated strains lacking the *vapA* gene or virulence plasmid. Image analysis suggested that the time-dependent cell response profile (TCRP) is governed by cell size and roundness as well as cytoxicity of infecting *R. equi* strains. The amplitude and inflection point of the resulting TCRP were dependent on the multiplicity of infection as well as virulence of the infecting strain, thus distinguishing between virulent and attenuated strains.

## Introduction

*Rhodococcus equi* is a parasite of macrophages, and the causative agent of pyogranulomatous pneumonia in young foals. In addition, *R. equi* infects other animals including pigs and cattle, and is an opportunistic human pathogen [1]. Following phagocytosis by macrophages, *R. equi* arrests phagosomal maturation. As a result, phagolysosomal fusion, the respiratory burst and acidification of the phagosomal compartment do not take place, thus providing *R. equi* with an intracellular compartment that allows its rapid growth. Eventually the macrophage is killed in a necrotic manner [2]–[6]. All virulent isolates harbour a plasmid containing a pathogenicity island, which is required for arresting phagosomal maturation and proliferation in macrophages [3], [7]. The virulence associated protein VapA encoded within the pathogenicity island, is essential, but not sufficient, for proliferation in macrophages [8]. In addition to the virulence plasmid encoded genes, other chromosomally encoded proteins have been shown to be required for virulence [9], [10].

The recent annotation of the *R. equi* genome [11] and the development of genetic tools for random [12], [13] and directed mutation [14], allows for a high throughput identification of virulence and virulence associated genes. Virulence of *R. equi* is commonly evaluated by enumerating intracellular *R. equi* levels using a variety of techniques, including staining of *R. equi* followed by microscopy or cytometry [6], [15], incorporation of ^3^H-uracil in macrophage monolayers [6], [16], lysis of macrophages followed by plating the lysate on agar plates to determine the number of colony forming units [10], or by determining the number of *R. equi* 16S rRNA genes using quantitative real-time PCR [17]. A major limitation of these methods is that they determine the number of intracellular bacteria at set time points and do so by killing the infected cell. These methods therefore do not provide real-time information regarding infection of living cells, are time consuming and do not readily lend themselves to high throughput analysis.

An impedance based method to analyse changes in cell morphology and physiology may overcome many of these drawbacks. In this system an alternating electrical current is passed through microelectrodes placed at the bottom of a cell culture well to detect changes in impedance, which relates to the resistance of a system to an alternating current. The presence of cells adhering to the surface of the well changes the impedance of the system, which is converted into the dimension-less cell index (C_i_) value, which is the output of the instrument. Cell morphology, viability and cell number are some of the parameters that affect the cell index. For example, the C_i_ value is increased when more cells adhere to the surface of the cell culture well, whereas a loss of viability leads to a decrease of the C_i_ value. A major advantage of this system is that it allows label-free analysis of the effects of experimental manipulation on the cell response in real-time [18]–[21]. Recently, an impedance based system was used to analyse the response of host cells to infection by the human pathogens *Neisseria meningitidis* and *Salmonella typhimurium* [22], [23].

The aim of this study was to determine whether an analysis of changes in impedance in a cell culture well can be applied to study infection of macrophages with *R. equi*, and whether it can distinguish between infection of macrophages with virulent or attenuated *R. equi* strains. The results of this study were underpinned by image analysis of infected macrophages. The data show that determination of changes in impedance in cell culture wells represents a novel methodology to study the interaction of *R. equi* with macrophages in real-time.

## Materials and Methods

### Bacterial strains and growth conditions

Bacterial strains and plasmids used in this study are listed in Table 1. *E. coli* was grown in Luria Bertani (LB) medium [24]; *R. equi* strains were grown in mineral medium supplemented with 20 mM acetate [25] or in brain heart infusion (BHI) broth at 37°C. Where appropriate, apramycin (30 µg ml^−1^ *E. coli* or 80 µg ml^−1^ *R. equi*) was added. For solid media agar was added (1.5% [w/v]).

**Table 1:** Bacterial strains, plasmids and oligonucleotides used in this study.

Name	Sequence or genotype	Reference
**Strains**		
*E. coli* DH5α	*supE*44 _ *lacU*169, (φ80*lacZ*_ M15) *hsdR*17 *recA*1 *endA*1 *gyrA*96 *thi-*1 *relA*1	Bethesda Research Laboratories
*R. equi* 103+	Virulent strain	[30]
*R. equi* 103−	Virulence plasmid free strain	This study
*R. equi* Δ*vapA*	*R. equi* 103+ Δ*vapA*	This study
*R. equi* Δ35000	*R. equi* 103+ ΔREQ35000	This study
**Plasmids**		
pSelAct	Apr^R^, *lacZ, codA:upp*	[14]
pVapAK	pSelAct derivative containing fragments upstream and downstream of *vapA*	This study
p35000K	pSelAct derivative containing fragments upstream and downstream of REQ35000	This study
**Oligonucleotides**		
VapA_1489AF	CCGTGAATTCTTGAGGATGTCGTGTCTTGC	This study
VapA_1489AR	GTCGTGATCATCGCCTTAGAAACCGTCTTG	This study
VapA_1412BF	ATAAGGATCCATATCCAGTCCGGTGGAGT	This study
VapA_1412BR	CGCTTCTAGACCAATCATTGTGCTGACACC	This study
35000A_1252F	ACGGTCTAGATCCTCGGTGTTCTTTCATCC	This study
35000A_1252R	AGCAGAATTCCAGAAGGATTCGCAGTTCGT	This study
35000B_1403F	GTCAGAATTCGCAAGGCTCAGAGGATGTCT	This study
35000B_1403R	TAATAGGGCCCGAGATCGAGAACACCGACT	This study
VapA678_1249F	GCATCTAGAAGACCAACATCGTTCGCG	This study
VapA678_1249R	CTAACATGTCTAGGCGTTGTGCCAGCTACCA	This study
35000E_229F	CACGCTACGGTTGTCCACTA	This study
35000E_229R	GTGGAGGGCGTACAGGTG	This study
vapA_182F	AATGCGACCGTTCTTGATTC	[17]
vapA_182R	TCTCCGTGAACGTCGTACTG	[17]
35000i_193F	GACGAGTTCGACGAGGTCAC	This study
35000i_193R	CATCTCTGATGCGGGTCTTC	This study
16SrRNA200F	ACGAAGCGAGAGTGACGGTA	[27]
16SrRNA200R	ACTCAAGTCTGCCCGTATCG	[27]

### DNA manipulations

Chromosomal DNA was isolated as previously described [26]. Plasmid DNA was isolated using the High Pure Plasmid Purification Kit (Roche) as described by the manufacturer. PCR with appropriate oligonucleotide primers (Table 1) was done using High Fidelity Phusion Hot Start Polymerase according to the manufacturer's recommendations (New England Biolabs). Other DNA manipulations were carried out using standard procedures [24].

### Construction of *R. equi* mutants

Primers and plasmids used in this study are listed in Table 1. Primer pair VapA_1489AF and VapA_1489AR and primer pair VapA_1412BF and VapA_1412BR were used to amplify the 5′ and the 3′ ends of *vapA* and their flanking regions to give rise to fragments vapA_A (1489 bp) and vapA_B (1412 bp). VapA_B was digested with BamHI and XbaI and ligated into the corresponding restriction sites of pSelAct. The resulting plasmid was digested with EcoRI and BamHI and ligated to the vapA_A fragment digested with the same enzymes to give pVapAK.

Primer pair 35000A_1252F and 35000A_1252R and primer pair 35000B_1403F and 35000B_1403R were used to amplify the 5′ and 3′ ends of REQ35000 and their flanking regions to give rise to fragments 35000A (1252 bp) and 35000B (1403 bp). The latter fragment was digested with EcoRI and ApaI and ligated into the corresponding sites of pSelAct. The resulting plasmid was digested with XbaI and EcoRI and ligated to 35000A that was digested with the same enzymes giving rise to p35000K.

Plasmids pVapAK and p35000K were introduced into *R. equi* via electroporation [13]. Transformants resulting from integration of the suicide vector into the genome were selected on LB agar plates containing apramycin. Apramycin resistant colonies were grown overnight in LB liquid medium without apramycin allowing a second recombination to take place, resulting in either restoration of the wild-type, or in an exchange of the wild type for the mutated gene. The culture was plated on mineral medium agar plates containing acetate and 5-fluorocytosine to select for strains that had lost pSelAct [14].

The genotype of the mutants was confirmed by amplifying an internal fragment of the deleted gene, which is absent in the mutant and present in the wild type strain. In addition, primers complementary to regions flanking the deletion were used, which gave a smaller fragment using genomic DNA from the mutant strain compared to the wild type. The primer pairs amplifying an internal fragment of *vapA* and REQ35000 were vapA_182F/vapA_182R and REQ35000i_193F/REQ35000i_193R, respectively. The primer pairs vapA678_1249F/vapA678_1249R and REQ35000E_229F/REQ35000E_229R were used to amplify fragments that encompassed the *vapA* and REQ35000 deletions, respectively.

A virulence plasmid-free derivative of *R. equi* 103+ was isolated by growing bacteria at 37°C in BHI. Subsequently dilutions of the culture were plated onto BHI agar plates and single colonies were analysed by PCR to detect the presence of the virulence plasmid.

### Macrophage infections

The macrophage-like cell line J774A.1 (American Type Culture Collection), cultured in DMEM supplemented with 10% (v/v) fetal bovine serum and 2 mM L-glutamine, was seeded in 24 well plates (6×10^5^ cells/ml, final volume 1 ml) and grown overnight in 37 °C with 5% CO_2_. Changes in impedance following infection of macrophages were determined using the xCELLigence system according to the manufacturer's instructions (Roche), using a 96-well tissue culture E-Plate (Roche) that was seeded with 4×10^5^ J774A.1 cells per well.

For image analysis a 96 well black microplate with a transparent bottom (Perkin Elmer) was seeded with 1.8×10^4^ J774A.1 cells/well using the MultiDrop 384 (Thermo Scientific). Bacteria grown in BHI were harvested in the exponential phase of growth and were washed twice with phosphate buffered saline (PBS). Macrophages were infected with *R. equi* for 1 hour; the monolayers were washed 3 times with PBS (37°C) and the medium was replaced with DMEM supplemented with 5 µg/ml vancomycin.

### Enumeration of macrophages

The trypan blue exclusion assay was employed to determine cell number and viability of the J774A.1 cells. Briefly, macrophage monolayers were evenly suspended in PBS. An aliquot of the cell suspension (20 µl) was mixed with an equal volume of 0.4% (v/v) trypan blue dye, and was incubated at room temperature for 5 min. Subsequently 20 µl of the mixture was loaded into both counting chambers of a haemocytometer. Cells stained in blue were considered not viable. Only counts with 20–50 cells per square were considered.

### Labelling of *R. equi* with ATTO 488

*R. equi* grown in BHI broth was washed twice with NaHCO_3_ buffer (100 mM, pH 8.3). Subsequently dilutions containing 1.5×10^8^ bacteria/ml in NaHCO_3_ buffer were prepared. Aliquots (1 ml) were stained with the ATTO 488 fluorophore (50 µg/ml) for 45 min at room temperature with gentle shaking. Bacteria were subsequently washed once with 20 mM Tris-HCl buffer (pH 8.0) followed by two washes with PBS.

### Labelling of J774A.1 cells

Infected monolayers were fixed with 4% (v/v) paraformaldehyde (PFA) for 20 min at room temperature and were subsequently permeabilized with 0.1% (v/v) Triton X-100 in PBS for 5 min with shaking. The detergent was removed by washing the monolayer three times with 150 µl PBS. The actin cytoskeleton was subsequently stained with Texas Red-X Phalloidin (1∶100 in PBS; Invitrogen) for 15 min at room temperature, followed by three washes with PBS to remove the unbound stain. Cell nuclei were stained with Hoechst 33258 (1∶5000 in PBS; Sigma Aldrich) for 15 min at room temperature. Monolayers were washed three times with PBS to remove unbound stain and were taken up in 150 µl PBS per well at 4°C until visualised.

### Fluorescence microscopy

Image acquisition of macrophage monolayers was performed on an Olympus ScanˆR automated screening microscope using a 40× FLN objective. Automated cell identification during imaging was object-based with a threshold pixel intensity of 600, with 200 as minimum and 100000 as maximum object size values. A minimum of 12 sub-positions per well were acquired. Hoechst, Texas Red phalloidin and ATTO 488 were imaged with standard filter sets using exposure times of 100 ms, 100 ms and 200 ms, respectively. Images were saved in non-compressed 12-bit format.

### Image analysis

High content analysis of images was carried out using Perkin Elmer Columbus software. The Texas Red Phalloidin channel was used for segmenting individual cells using standard algorithms within the software. Cell masks based on each segmented cell were then used to calculate cellular features including cell area and roundness, and in addition the number of *R. equi*-positive structures per cell was determined.

### Enumeration of intracellular *R. equi*

Intracellular proliferation of *R. equi* was assessed by real-time qPCR. Monolayers washed twice with PBS were scraped, spun down and resuspended in 10 mM Tris-HCl, pH 8.0. Harvested samples were heated at 99°C for 10 min, and then spun down briefly to pellet cellular debris. Real time-qPCR amplifying 16S rRNA was performed using oligonucleotides 16SrRNA200F and 16SrRNA200R as described previously [27]. Cycle threshold values were used to calculate the *R. equi* cell number using a standard curve of known amounts of bacteria with *r* ^2^ coefficients larger than 0.9952 in the range of 1×10^2^ to 1×10^6^ bacteria per reaction.

## Results

### The time-dependent cell response profile (TCRP) of infected J774A.1 monolayers depends on the multiplicity of infection

**Figure 1:**
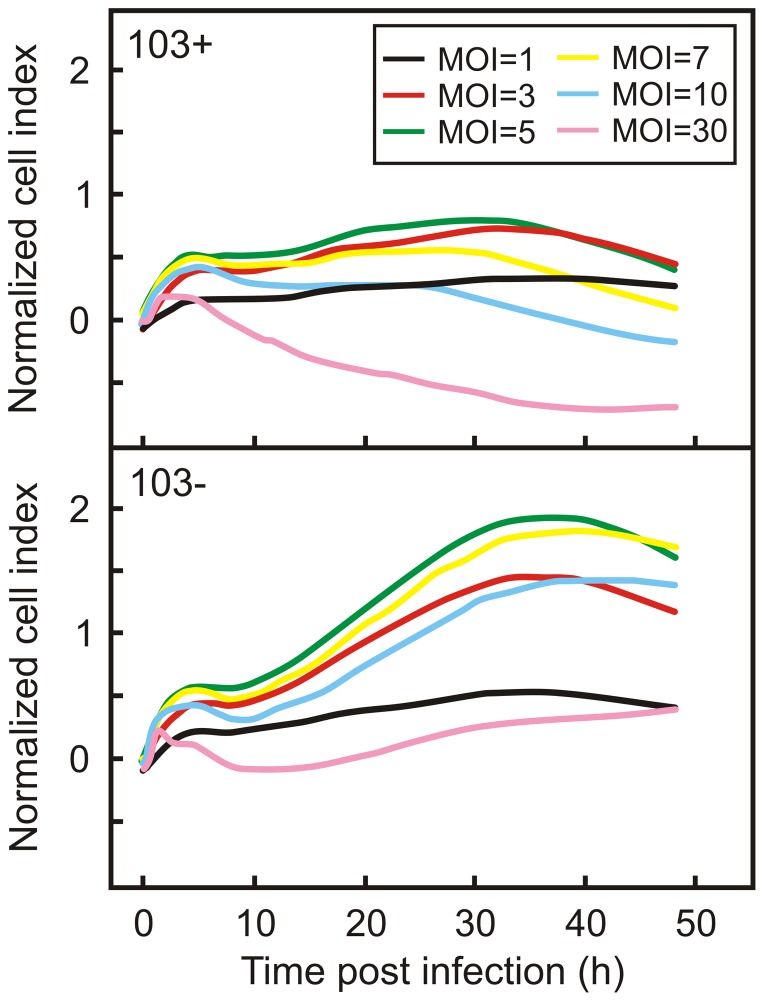
Time-dependent Cell Response Profile (TCRP) of infected macrophage monolayers. Monolayers were infected with a virulent (*R. equi* 103+; top panel) or with an avirulent (*R. equi* 103−; bottom panel) strain of *R. equi* at increasing multiplicities (MOI) of infection. The TCRP was normalized against the TCRP of a non-infected macrophage monolayer.

To determine the effects of infection on the behaviour of macrophages, J774A.1 cells were infected with *R. equi* 103+ using increasing multiplicities of infection (MOI) ranging from 1 to 30 bacteria per cell. Following infection, the change in cell index (C_i_) was recorded for 48 hours. The resulting time-dependent cell response profiles (TCRPs) were normalized against the profile of non-infected macrophage monolayers (Fig. 1).

**Figure 2:**
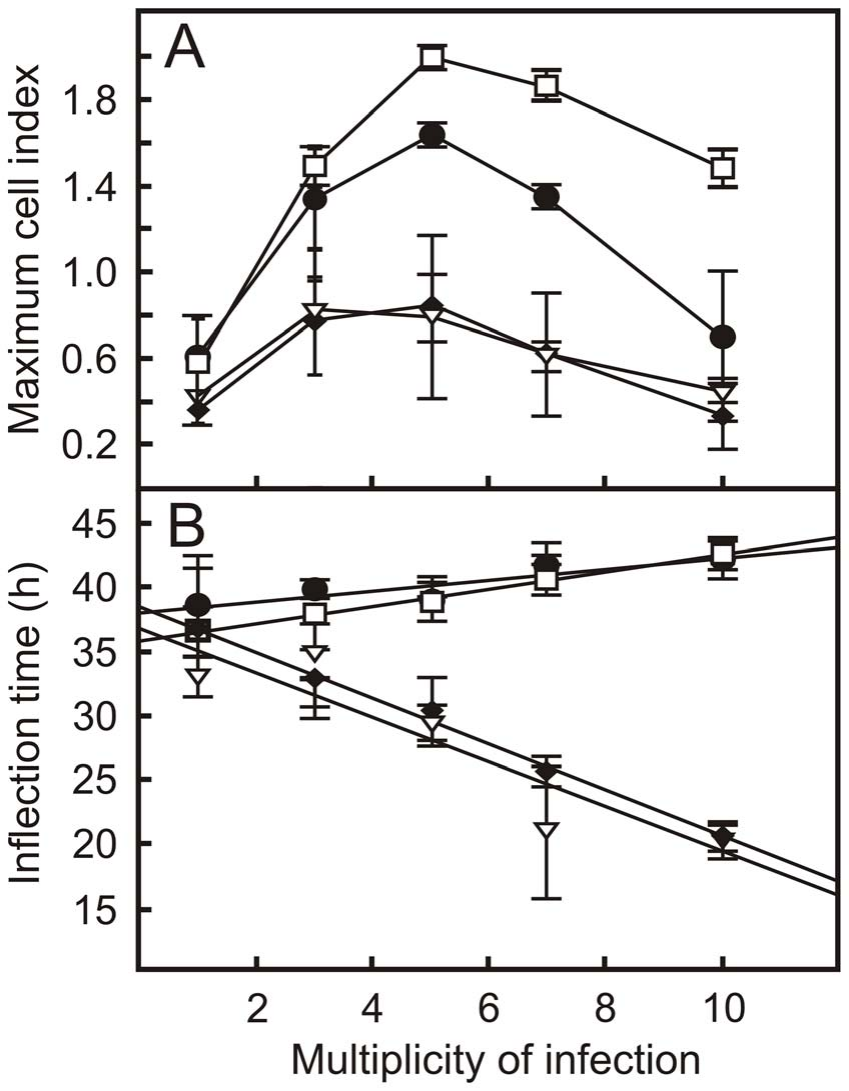
Key TCRP parameters of macrophage monolayers infected with virulent or attenuated *R. equi* strains. Shown are the maximum cell indices (Ci) values (panel A) and the the C_i_ inflection times (panel B) following infection of J774A.1 macrophage monolayers with virulent (♦, *R. equi* 103+; ∇, *R. equi* Δ35000) and attenuated (□, *R. equi* 103−; •, *R. equi* Δ*vapA*) *R. equi* strains at increasing multiplicity of infection. Values are the means ± SD.

Two TCRP parameters were of particular interest: the amplitude of the profile, which is the maximum C_i_ value, and the inflection time, which is the time when the highest C_i_ value in the TCRP profile is recorded. Interestingly, both parameters were dependent on the MOI used in the experiment. The highest C_i_ value was recorded when cells were infected with a MOI of 5 (Fig. 1; Fig. 2a). In contrast, the inflection time decreased with increasing MOI (Fig. 2b). Macrophages thus clearly respond to infection with virulent *R. equi* as determined by changes in impedance measured by the xCELLigence system.

### Phenotypic differentiation of macrophages infected with virulent and attenuated *R. equi*

**Figure 3:**
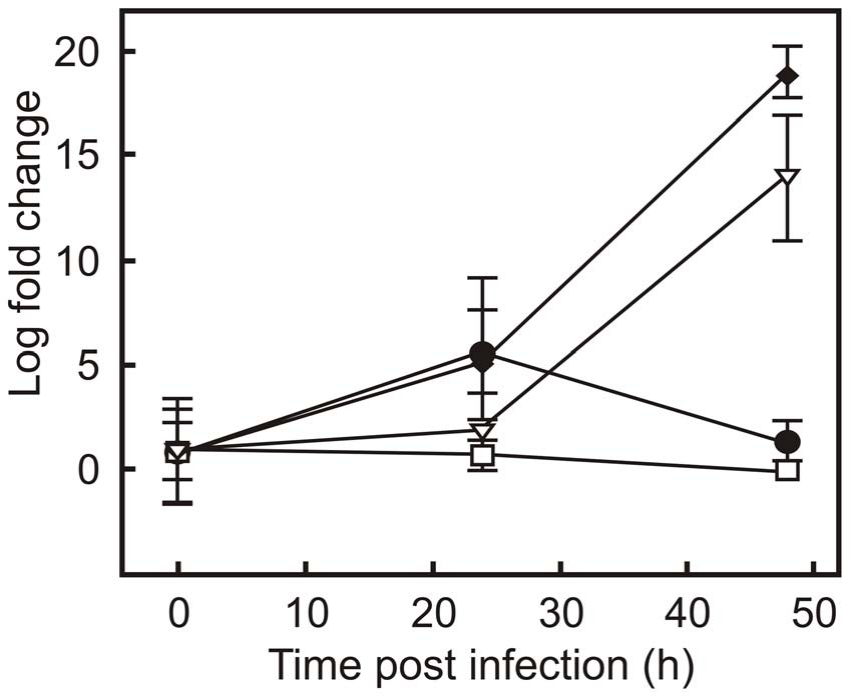
Intracellular growth of virulent and attenuated *R. equi*. J774A.1 monolayers were infected with virulent (♦, *R. equi* 103+; ∇, *R. equi* Δ35000) and attenuated (□, *R. equi* 103−; •, *R. equi* Δ*vapA*) *R. equi* strains. Following a 1 hour incubation to allow phagocytosis, monolayers were washed and treated with vancomycin to kill remaining extra cellular bacteria. Intracellular bacteria were enumerated via real time qPCR of the *R. equi* 16S rRNA gene. Results are expressed as fold change in bacterial numbers relative to t = 0 (h) values. Error bars denote the standard deviation of the mean.

The intracellular compartments in which attenuated strains of *R. equi* reside are different from those that contain virulent *R. equi* [2]–[5]. It is therefore conceivable that these differences in macrophage response to infection with virulent or attenuated strains of *R. equi* may be reflected in differences in the TCRP. To examine this, macrophage monolayers were infected with the attenuated strains strain *R. equi* 103− and *R. equi* Δ*vapA*. The former strain lacks the virulence plasmid, whereas the virulence factor *vapA* was deleted in the latter strain. In addition, strains were infected with the wild type strain and *R. equi* Δ35000, both of which are virulent. REQ35000, which was deleted in the latter strain, encodes an alternative sigma factor of RNA polymerase [11], which is not required for growth in macrophages (Fig. 3). *R. equi* Δ35000 was included in this experiment to contrast the results obtained with *R. equi* Δ*vapA*. In the latter strain a virulence factor encoding gene is deleted, whereas in the former a gene was deleted that does not play a role in virulence. As expected, the virulent strains grew within J774A.1 macrophages, whereas the attenuated strains did not (Fig. 3). The TCRP profiles of virulent *R. equi* and attenuated strains differed considerably. The amplitude of the TCRP profile was dependent on the MOI when monolayers were infected with either virulent or attenuated strains, with maximum amplitude occurring at a MOI of 5. However, the amplitude was significantly higher when monolayers were infected with attenuated strains at all MOI (Fig 1; Fig 2a). The inflection time of the profile was positively correlated to the MOI when macrophages were infected with attenuated strains, whereas a negative correlation was observed when virulent strains were used (Fig. 2b). The xCELLigence system thus revealed major differences in macrophage responses to infection with either virulent or attenuated *R. equi* strains.

### Infected macrophage monolayers are not homogeneous

**Figure 4:**
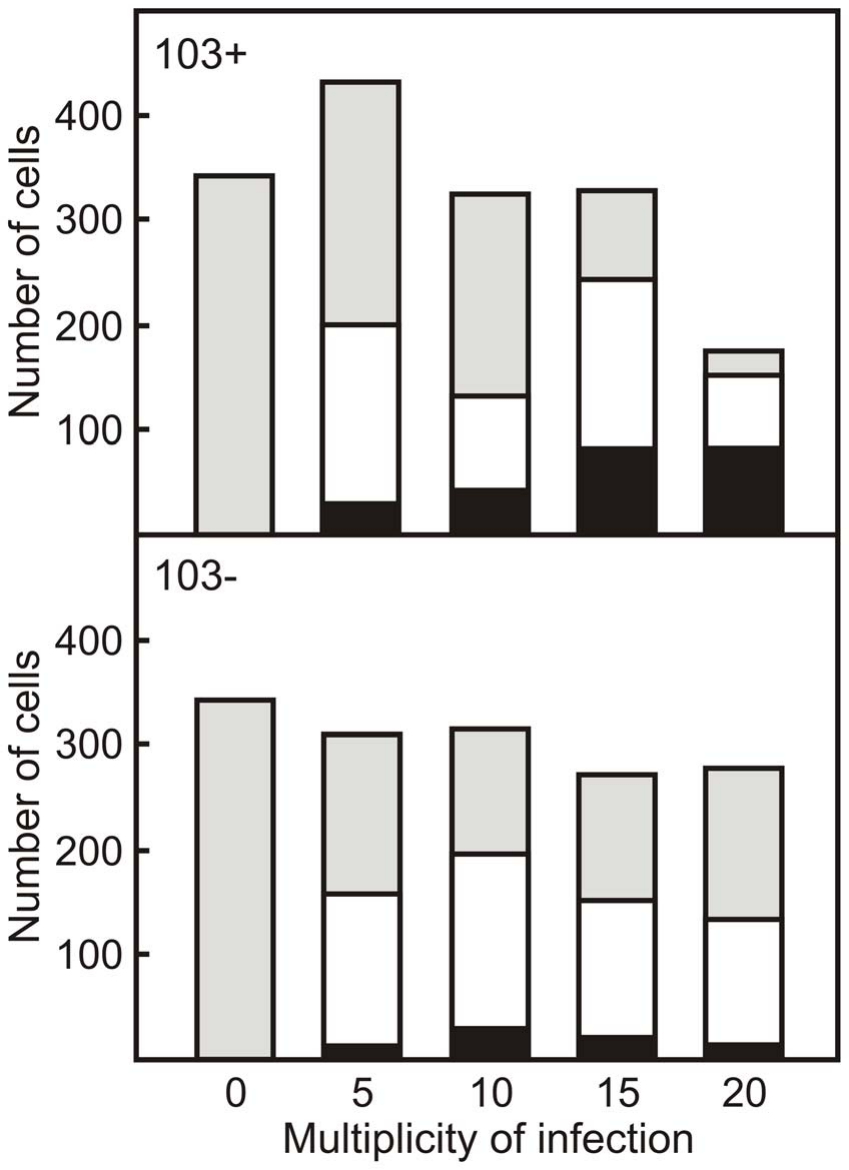
Macrophages in infected monolayers are not homogeneous with respect to the number of intracellular *R. equi* they harbour. Macrophage monolayers were infected with ATTO 488 labeled virulent (*R. equi* 103+; top panel) or attenuated (*R. equi* 103−; bottom panel) strains of *R. equi* at increasing multiplicities (MOI) of infection. Monolayers were fixed and labelled 24 h post-infection. High content image analysis using Columbus software was employed to enumerate infected macrophages and to determine the number of intracellular *R. equi*. Shown are the number of macrophages that contained no intracellular bacteria (grey), between 1 and 5 bacteria (white) or more than 5 bacteria (black).

The TCRP profile of a monolayer is dependent on a number of parameters including cell number, cell size and cell morphology. To get an insight into why the TCRP of monolayers infected with virulent *R. equi* strains differ significantly from those that were infected with attenuated strains, a detailed image analysis of individual cells was carried out. The number of J774A.1 cells remained constant in monolayers infected with either the virulent wild type *R. equi* 103+ strain or the attenuated plasmid free *R. equi* 103− strain. The only decrease in cell numbers was observed when monolayers were infected with the virulent strain at a MOI = 20 (Fig. 4). There are therefore no major differences in cell numbers that would explain the observed differences in TCRP of monolayers infected with virulent or attenuated strains.

The macrophage population in infected monolayers could be divided into three groups based on the number of intracellular bacteria: a group containing no intracellular bacteria, a group that contained 1 to 5 bacteria, and a group that harboured more than 5 intracellular bacteria. The number of cells within each of these groups remained constant at increasing MOI when the monolayer was infected with the attenuated *R. equi* 103− strain. However, the proportion of infected cells increased dramatically following infection of monolayers with virulent strains at increasing MOI. In particular the proportion of macrophages containing 5 or more *R. equi* increased significantly (P<0.05; Fig. 4). These data thus show that infected monolayers are not homogeneous in terms of number of intracellular bacteria present, and that major differences exist between monolayers that are infected with virulent or attenuated strains.

### Infection of macrophages with *R. equi* affects cell morphology

**Figure 5:**
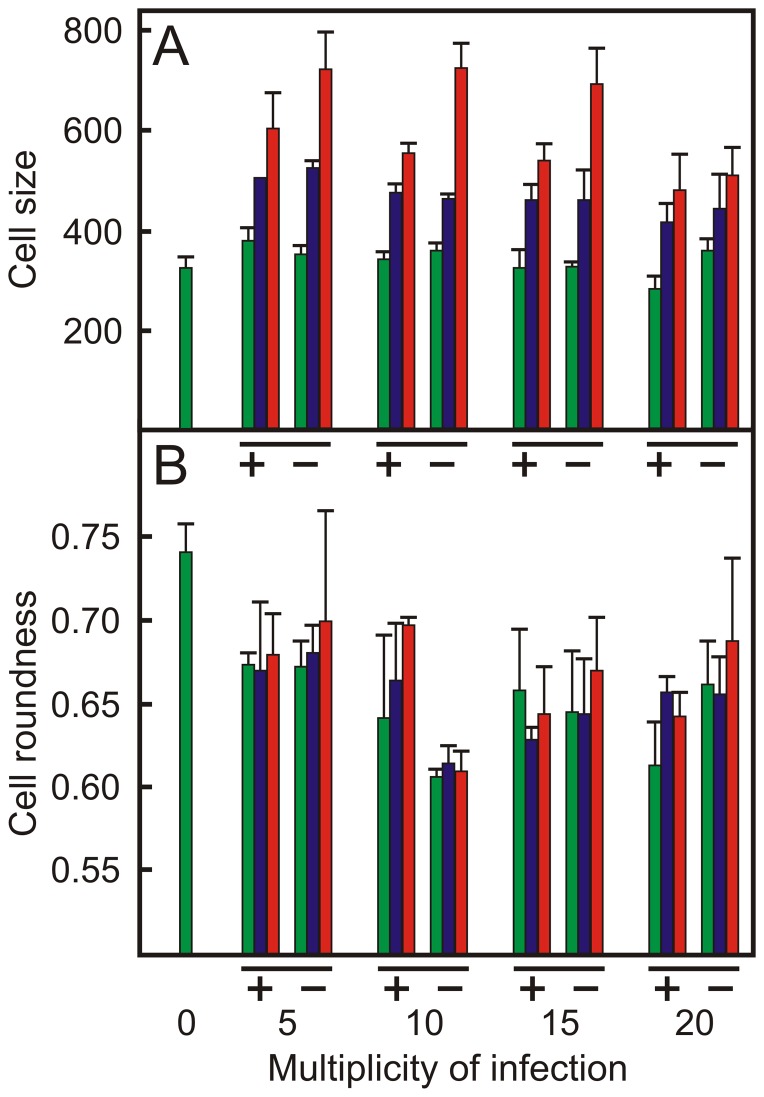
Infection of macrophages with *R. equi* affects host cell size and morphology. Macrophage monolayers were infected with ATTO 488 labeled virulent *R. equi* 103+ or attenuated *R. equi* 103− strains at increasing multiplicities (MOI) of infection. Monolayers were fixed and labelled 24 h post-infection. The cell size (panel A) and cell roundness (panel B) of infected cells were compared to control cells (not infected, indicated as MOI = 0). Green bars: no intracellular bacteria; blue bars: 1 to 5 intracellular bacteria; red bars: more than 5 intracellular bacteria. +: *R. equi* 103+; −: *R. equi* 103−. Error bars denote the standard deviation of the mean.

**Figure 6:**
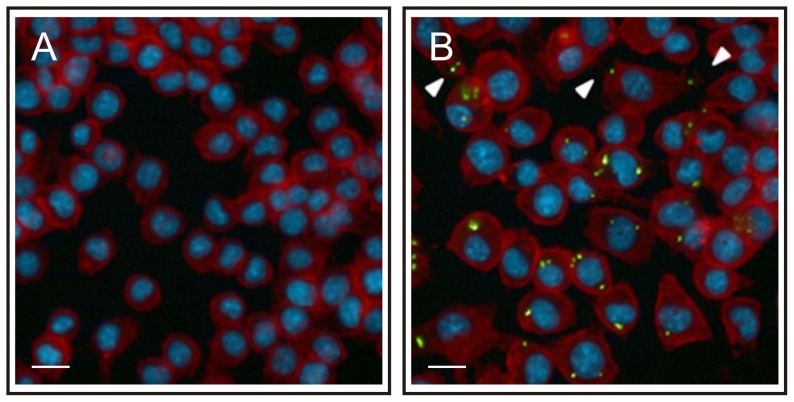
The morphology of macrophages changes following infection with *R. equi*. J774A.1 monolayers were infected with ATTO 488 labelled virulent *R. equi* 103+ (green). Monolayers were fixed 24 h post-infection; the actin cytoskeleton and cell nuclei were stained with Texas Red Phalloidin (red) and Hoechst 33258 (blue), respectively. Panel A: non-infected monolayers, panel B: monolayers infected with *R. equi* 103+. Arrows indicate the change in cell shape following infection with *R. equi*. The length of the white bar is 40 µm.

The morphology of macrophages in monolayers infected with the wild type strain *R. equi* 103+ or the attenuated strain *R. equi* 103− was subsequently determined using fluorescence microscopy and high content image analysis and compared to that of cells present in non-infected monolayers (Fig. 5, Fig. 6). The cell size increased with increasing numbers of intracellular bacteria at all multiplicities of infection. Cells that did not contain intracellular bacteria were of the same size as those present in non-infected monolayers. Cells that contained 5 or more intracellular bacteria were larger in monolayers infected with the attenuated strain than those present in monolayers infected with virulent *R. equi* (Fig. 5a).

The roundness of macrophages in infected monolayers decreased significantly (P<0.05) in comparison to cells present in non-infected monolayers (Fig 5b, Fig 6). In contrast to the cell size, the roundness of cells in infected monolayers that did not contain intracellular bacteria decreased to the same extent as those that harboured intracellular bacteria. Infection with virulent or attenuated *R. equi* had a similar impact on cell roundness (Fig. 5b).

## Discussion

The recent annotation of the *R. equi* genome has provided a fascinating insight into the evolution of this pathogen. It showed that chromosomal genes that are shared with non-pathogenic actinomycetes were co-opted to support a virulent lifestyle of the ancestor of *R. equi* following acquisition of the virulence plasmid [11]. A major challenge of the *R. equi* post-genomic era is to identify those genes that play a role in virulence. Although efficient mutagenesis tools for *R. equi* have been developed [12]–[14], the analysis of the resulting mutants with regard to their virulence is time consuming. The aim of this work was to demonstrate that measurement of impedance changes in cell culture wells using the xCELLigence system [22], [23], [28] can be used as a high-throughput system to analyse virulence of *R. equi* in real-time and that any effects recorded could be correlated to visual changes in the host cells.

Image analysis of infected monolayers showed that the macrophage population is not homogeneous in relation to the numbers of intracellular bacteria following infection with either wild type or attenuated strains. A large number of cells harboured no intracellular bacteria, whereas others harboured 5 or more *R. equi*. This heterogeneity in infection levels of individual cells has been observed previously [6]. Infection of macrophages with *R. equi* had a profound impact on their morphology: they increased in size and became less round. There was no difference in morphology between macrophages that were infected with the virulence plasmid containing *R. equi* 103+ or with the attenuated plasmid free strain *R. equi* 103−, which agrees with earlier observations [15]. Macrophages infected with *R. equi* produce IL-1β, IL-6, IL-10, IL-12 p40 and TNF-α [15], which affect cell morphology [29]. Interestingly, this study showed that non-infected cells within infected monolayers displayed similar reductions in cell roundness as infected cells. In contrast, the cell size of macrophages in infected monolayers that did not contain intracellular bacteria was the same as those in non-infected monolayers. Cells became larger as they contained more intracellular bacteria. This suggests that cytokines produced by infected cells affect the roundness, but not the size of non-infected cells.

These morphological changes increase the well area covered by macrophages and hence increase the impedance. Furthermore J774A.1 macrophages continue to grow, also resulting in an impedance increase. This is reflected in the TCRP profiles, which showed an increase in C_i_ value following macrophage infection. *R. equi* is cytotoxic to macrophages, resulting in cell death and release of macrophages from the well surface [3], [6]. *R. equi* cytoxicity is particularly apparent 24–48 hours post infection, which probably accounts for the decrease in C_i_ value towards the end of the macrophage infection analysis.

The maximum C_i_ or amplitude of the TCRP was dependent on the MOI, with a maximum C_i_ at MOI = 5 for monolayers infected with either the virulence plasmid containing or attenuated virulence plasmid free strain. However, the amplitude of the TCRP of macrophages infected with a plasmid-free attenuated strain of *R. equi* was at least twice as high as that of cells infected with the virulence plasmid harbouring virulent strains. Changes in cell morphology (size and roundness) following infection leading to an increase in C_i_ were the same regardless whether the cell was infected with the virulent wild type or the attenuated plasmid-free strain. However, although both strains are cytotoxic, strains that harbour the virulence plasmid are at least 8 times more cytotoxic than virulence plasmid free strains [3], [6]. Cytotoxity becomes increasingly important at high MOI [3], thus explaining both the lower amplitude as well as the negative correlation between MOI and inflection time of the TCRP of macrophages infected with the virulent strains.

Deletion of the *vapA* gene impairs the ability of *R. equi* to arrest phagosomal maturation as is the case in the virulence plasmid-free strain. *R. equi* Δ*vapA* is therefore completely attenuated [4], [8]. However, deletion of *vapA* does not abolish cytotoxicity of the wild type strain [4]. This intermediary phenotype is reflected in the TCRP of macrophage monolayers infected with *R. equi* Δ*vapA*, which had a high C_i_ value at MOI = 5 comparable to that of the attenuated virulence plasmid-free strain, yet a strongly reduced C_i_ value at MOI = 10, comparable to that of the wild type strain.

In conclusion, this work demonstrates that the measurement of changes in impedance in cell culture wells using the xCELLigence system is a powerful tool to analyze the interaction between *R. equi* and macrophages in real-time. Changes in impedance can be correlated to visual data coming from high content analysis, reflecting changes in macrophage morphology following infection. In addition, this system lends itself to a high-throughput approach to identify mutants affected in virulence, and thus is highly useful in identifying virulence (associated) factors in the *R. equi* post-genomic era.
